# Improvement of Functional Properties of Wheat Gluten Using Acid Protease from *Aspergillus usamii*

**DOI:** 10.1371/journal.pone.0160101

**Published:** 2016-07-28

**Authors:** Lingli Deng, Zhaoxia Wang, Sheng Yang, Junmei Song, Fei Que, Hui Zhang, Fengqin Feng

**Affiliations:** 1 College of Biosystems Engineering and Food Science, Fuli Institute of Food Science, Zhejiang Key Laboratory for Agro-Food Processing, Zhejiang R & D Center for Food Technology and Equipment, Zhejiang University, Hangzhou 310058, China; 2 Shanghe Comprehensive Testing Center, Jinan 251600, China; 3 School of Food and Bioengineering, Qilu University of Technology, Jinan 250353, China; 4 Department of Applied Engineering, Zhejiang Economic and Trade Polytechinc, Hangzhou 310018, China; National University of Ireland - Galway, IRELAND

## Abstract

Hydrolysis parameters (temperature, E/S ratio, pH, and time) for acid protease (from *Aspergillus usamii*) hydrolysis of wheat gluten were optimized by response surface methodology (RSM) using emulsifying activity index (EAI) as the response factor. A temperature of 48.9°C, E/S ratio of 1.60%, pH 3.0, hydrolysis time of 2.5 h was found to be the optimum condition to obtain wheat gluten hydrolysate with higher EAI. The solubility of wheat gluten was greatly improved by hydrolysis and became independent of pH over the studied range. Enzymatic hydrolysis resulted in dramatically increase in EAI, water and oil holding capacity. Molecular weight distribution results showed that most of the peptides above 10 kDa have been hydrolyzed into smaller peptides. The results of FTIR spectra and disulfide bond (SS) and sulfhydryl (SH) content suggested that a more extensional conformation was formed after hydrolysis, which could account for the improved functional properties.

## Introduction

Wheat gluten is an economically important coproduct in the recovery of wheat starch in wet milling of wheat flour. With the expansion of wheat starch production, the utilization of wheat gluten is getting diversified. However, the poor water solubility of wheat gluten hinders its application as a functional ingredient in food industry. Even though the price of wheat gluten is lower compared to that of protein isolates from animal sources, it has a narrower range of industrial applications [1]. In order to broaden the utilization of wheat gluten, the functional properties such as solubility and emulsifying capacity need to be improved. A lot of work has been done to improve the functional properties by physical [2, 3], chemical [4, 5], and enzymatic modifications [1, 6, 7].

Enzymatic hydrolysis has been studied intensively because of the advantages in specificity, high-efficiency, and low energy consumption. An early study reported that the foaming property of gluten was compared to that of egg white protein after mild enzymatic treatments (pepsin + papain), and the emulsifying property was improved dramatically after combined treatment (deamidation + pepsin + papain) [6]. It was found that the emulsifying and foaming properties of alcalase-assisted hydrolysates with a moderate degree of hydrolysis (DH) of 5.0% were remarkably higher than the native gluten [8]. In a recent research, the gluten peptides with a DH of 3.0% by alcalase and trypsin showed excellent emulsifying properties [7]. Studies have been conducted to reveal how processing parameters affected the functional properties of wheat gluten during enzymatic hydrolysis. Different proteases have been used in previous studies such as pepsin, trypsin, papain, chymotrypsin, and alcalase, but little was known about the hydrolysis effect of acid protease from *Aspergillus usamii* (EC 3. 4. 23. 18) on wheat gluten [1, 6, 9, 10].

The objective of this study was to obtain wheat gluten hydrolysate with improved emulsifying activity by acid protease hydrolysis, and characterize the functional properties of the hydrolysate. Hydrolysis parameters (hydrolysis temperature, enzyme-substrate ratio, pH, and hydrolysis time) on emulsifying activity were optimized using Box-Behnken response surface methodology. For the prepared wheat gluten hydrolysate, molecular distribution, Fourier transform infrared spectroscopy (FTIR), and disulfide bonds (SS) and sulfhydryl (SH) content were analyzed to reveal the structural changes after hydrolysis.

## Materials and Methods

### Materials

Wheat gluten (72.6% protein content) was supplied by Ruixiang Biotechnology Co., Ltd (Shandong, China). Acid protease (*Aspergillus usamii* No. 537, 50000 U/g, EC 3. 4. 23. 18) was purchased from Wuxi Enzymes (Jiangsu, China). Soybean oil was purchased from a local supermarket. DTNB (5,5'-Dithiobis-(2-nitrobenzoic acid)) and standard molecular markers for HPLC analysis (thyroglobulin, 670 kDa; ferritin, 440 kDa; BSA, 67 kDa; ovalbumin, 43 kDa; cytochrome C, 13.6 kDa; aprotinin, 6.5 kDa and vitamin B12, 1.4 kDa) were purchased from Sigma Aldrich (St. Louis, MO, USA). All other chemicals and reagents were purchased from Sinopharm Chemical Reagent Co., Ltd., China. The water used was double distilled.

### Experimental design and statistical analysis

Box-Behnken RSM was used to study the effect of four independent variables X_1_ (hydrolysis temperature, °C), X_2_ (enzyme-substrate ratio, E/S, w/w), X_3_ (pH), X_4_ (hydrolysis time, h) at three levels on the emulsifying activity (S1 Table). Preliminary experiments were carried out to determine the levels of the factors. The experimental design was shown in Table 1.

**Table 1:** Box-Behnken arrangement for independent variables of X_1_ (hydrolysis temperature, °C), X_2_ (E/S, w/w), X_3_ (pH) and X_4_ (hydrolysis time, h), and their response emulsifying activity index (EAI, m^2^/g).

Run order	Levels of independent variables				EAI (m^2^/g)
	X_1_ (hydrolysis temperature, °C)	X_2_ (E/S, w/w)	X_3_ (pH)	X_4_ (hydrolysis time, h)	
1	50 (0)	1.0 (-1)	3.5 (0)	2.5 (1)	52.14
2	50 (0)	1.5 (0)	4.0 (1)	1.5 (-1)	52.69
3	50 (0)	1.5 (0)	4.0 (1)	2.5 (1)	53.80
4	45 (-1)	2.0 (1)	3.5 (0)	2.0 (0)	49.56
5	50 (0)	2.0 (1)	3.0 (-1)	2.0 (0)	54.54
6	50 (0)	1.5 (0)	3.0 (-1)	1.5 (-1)	53.25
7	50 (0)	1.0 (-1)	4.0 (1)	2.0 (0)	50.39
8	55 (1)	1.5 (0)	3.5 (0)	2.5 (1)	55.09
9	50 (0)	2.0 (1)	4.0 (1)	2.0 (0)	47.90
10	50 (0)	1.5 (0)	3.5 (0)	2.0 (0)	55.64
11	50 (0)	1.5 (0)	3.5 (0)	2.0 (0)	54.90
12	55 (1)	1.0 (-1)	3.5 (0)	2.0 (0)	47.72
13	55 (1)	1.5 (0)	4.0 (1)	2.0 (0)	49.10
14	50 (0)	2.0 (1)	3.5 (0)	1.5 (-1)	51.22
15	50 (0)	1.5 (0)	3.5 (0)	2.0 (0)	56.19
16	45 (-1)	1.5 (0)	3.0 (-1)	2.0 (0)	57.48
17	50 (0)	1.5 (0)	3.5 (0)	2.0 (0)	54.90
18	55 (1)	1.5 (0)	3.5 (0)	1.5 (-1)	47.90
19	50 (0)	1.5 (0)	3.0 (-1)	2.5 (1)	58.77
20	50 (0)	2.0 (1)	3.5 (0)	2.5 (1)	54.17
21	45 (-1)	1.0 (-1)	3.5 (0)	2.0 (0)	47.35
22	50 (0)	1.0 (-1)	3.5 (0)	1.5 (-1)	49.19
23	55 (1)	1.5 (0)	3.0 (-1)	2.0 (0)	51.03
24	50 (0)	1.5 (0)	3.5 (0)	2.0 (0)	56.19
25	45 (-1)	1.5 (0)	3.5 (0)	1.5 (-1)	50.11
26	50 (0)	1.0 (-1)	3.0 (-1)	2.0 (0)	53.61
27	45 (-1)	1.5 (0)	4.0 (1)	2.0 (0)	49.38
28	45 (-1)	1.5 (0)	3.5 (0)	2.5 (1)	54.35
29	45 (-1)	2.0 (1)	3.5 (0)	2.0 (0)	49.38

Statistical analysis was performed using Design expert 8.0.6 software (Stat Ease, Inc., United States). For each hydrolysis parameter the variance was partitioned into three components (linear, quadratic, and interaction) to assess the adequacy of the second order polynomial function and relative importance of these components. The statistical significance of each model term was verified at a probability of *p* < 0.05. The adequacy of the model to navigate the design space of the responses was determined using the coefficient of determination (R^2^) and a lack of fit test. 3D response surface plots for the responses were generated for two independent variables while fixing the remaining variable at coded zero levels.

### Preparation of wheat gluten hydrolysates

Suspensions of 15% wheat gluten (w/v) were pre-incubated at a designated temperature (Table 1) in a water bath for 15 min. The pH was adjusted to a designated value and acid protease was added (w/w, based on protein content). The enzymatic hydrolysis was allowed to proceed for a designated time (Table 1). During reaction, the pH was monitored and maintained by adding drops of 3 M HCl. Hydrolysis was stopped by boiling the mixtures for 10 min. The mixtures were then centrifuged at 3,000 g for 10 min and the supernatant was spray dried for further analysis.

### Solubility

Samples were dispersed in pH 4, 5, 6, 7, 8 phosphate buffers (0.01M). The sample dispersions (1%, w/v) were vibrated in a shaker at 30°C for 30 min to allow complete hydration before centrifugation at 4000 g for 10 min at 4°C. The protein concentration of supernatant was determined by Kjeldahl method [11].

### Functional properties

The emulsion activity index (EAI) was determined by the method of Pearce and Kinsella [12] with modification. To prepare the emulsion, 15 ml of soybean oil and 45 ml of protein solution (0.1%, w/v) in 0.2 M phosphate buffer (pH 7.0) were mixed together in a 100 ml beaker and homogenized using a high-speed homogenizer unit for 1 min at 10,000 rpm. Aliquots of freshly prepared emulsion (100 μl) was taken from the bottom of the beaker and diluted with 5 ml of the 0.1% (w/v) sodium dodecyl sulfate (SDS) solution. The absorbance of the diluted emulsion was determined at 500 nm against blank (0.1% SDS solution). The emulsions were kept undisturbed for 10 min at room temperature before 100 μl of aliquots were taken from the bottom of the beaker and diluted with 5 ml of the 0.1% (w/v) SDS solution. The absorbance was measured, and EAI (m^2^/g) and ESI (min) was calculated as follows:

$$\text{EAI}\;[{\text{m}}^{2}/\text{g}]=2\left(2.303\times A_{0}\right)N\times 10^{-4}/\;\psi \text{LC}$$

where A_0_, absorbance of fresh emulsion; ψ, volume fraction of dispersed phase; L, light path in meters; C, concentration of protein; N, diluted folds.

$$\text{ESI}=A_{0}\times \Delta T/\left(A_{0}-A_{10}\right)$$

where A_0_, absorbance of fresh emulsion; A_10_, absorbance after 10 min; Δ*T*, 10 min.

Water holding capacity (WHC) was assayed using the modified method of Yamazaki *et al*. [13]. A 0.5 g of sample was transferred to a 15 ml polypropylene centrifuge tube with screw-cap. For each tube, 10 ml of distilled water was added, and centrifuged at 4000 g for 20 min at 4°C after 30 min vibration in a shaker at 25°C. Then, excess water was decanted and the tubes were inverted and left to drain for 10 min until it was weighed. The amount of water held was calculated by subtracting the weight before water addition and was expressed as wet weight basis.

Determination of oil holding capacity was conducted as described above, but distilled water was replaced by soybean oil.

### Degree of hydrolysis

The degree of hydrolysis was determined according to the method of Sorensen’s formol titration with modifications. 5 ml of neutralized formalin solution and 2 drops of indicator phenolphthalein were added to a 10 ml of 1% sample solution, and titrated with 0.1M NaOH. The end point is observed when the color just turns pink. The degree of hydrolysis (DH) was calculated from the volume of NaOH consumed, as given below:

$$\text{DH}\%=\frac{C_{0}\times \left(V_{1}-V_{2}\right)\times 1000}{C_{1}\times \text{V}\times h_{\text{tot}}}\times 100$$

where C_0_, the concentration of NaOH in mol/l; V_1_, the hydrolyzed wheat gluten consumed volume of NaOH in ml; V_2_, the unhydrolyzed wheat gluten consumed volume of NaOH in ml; C_1_, the concentration of sample in g/l; V, the volume of sample solution in ml; *h*_tot_, total number of peptide bonds in the protein substrate (8.38 meqv/g gluten protein).

### Molecular size distribution analysis

Molecular size distributions of the wheat gluten and its hydrolysate were determined by HPLC (Water’s 2695, USA) equipped with a G2000SWXL column (7.8 × 300 mm, TSK, Japan) and a diode array detector (Water’s 2996, USA). All the solutions were first filtered with a 0.4 μm filter and eluted with acetonitrile-water-trifluoroacetic acid (45:55:0.1) solution. The flow rate was 0.5 ml/min. The column temperature was 30°C, and detection wavelength was set at 220 nm. Standard molecular markers were used to calculate the molecular weight of samples.

### FTIR spectra

The FTIR spectra of samples were obtained using a Bruker Vector 22 (Bruker, German) with DTGS (deuterated triglycine sulfate) detector. Scanning was carried out in the range of 4000–400 cm^-1^ with the resolution of 4 cm^-1^, and 16 sans were applied for each sample. Deconvolution of infrared spectra was performed using Peakfit, Version 4.12 (SeaSolve Software Inc., United States). Band assignments in the amide I region (1600–1700 cm^-1^) were detected according to previous literatures [14]. Quantitative analysis of secondary structure components was performed using Gaussian peaks and curve fitting models according to the method of Byler and Susi [15]. All FTIR experiments were performed in triplicate.

### Determination of the SH and SS content

The disulfide bond (SS) and sulfhydryl (SH) contents were determined using DTNB (5,5'-Dithiobis-(2-nitrobenzoic acid)) according to the method of Zhao *et al*. [16]. For accessible free sulfhydryl content determination, 500 μl of the protein solution (1%, w/v) was mixed with 500 μl of buffer containing 8 M urea, 1% SDS, and 3 mM EDTA, 0.2 M Tris-glycine, pH 8.0. After adding 20 μl of DTNB, the reaction mixture was kept at 40°C for 15 min in a water bath to allow unfolding of protein. The solution was shaken at room temperature for 1 h. Absorbance was then measured at 412 nm (ε = 13,600 M^-1^ cm^-1^) and used to calculate accessible free SH content. The solution prepared as above without addition of sample was used as blank.

For determination of accessible total sulfhydryl (SH + reduced SS) content, a reaction buffer used was consisted of 8 M urea, 3 mM EDTA, 1% SDS, and 0.2 M Tris-HCl (pH 9.5), with 0.1 M Na_2_SO_3_ and 0.5 mM 2-nitro-5-thiosulphobenzoate (NTSB^2-^) synthesized from DTNB. The total sulphhydryl determination was conducted as described above. The content of SS was calculated as follows:

SS (μmol/g) = (total sulphhydryl content−free sulfhydryl content)/2

## Results and Discussion

### RSM analysis

RSM analysis has been applied to study the processing parameters of hydrolysis [17, 18]. Emulsifying activity, as the most widely used functional property to compare protein hydrolysates, is the response factor in this study [1, 8, 19, 20]. The Box-Behnken arrangement for independent variables of X_1_ (hydrolysis temperature, °C), X_2_ (E/S, w/w), X_3_ (pH), and X_4_ (hydrolysis time, h), and their response emulsifying activity index (EAI, m^2^/g) were shown in Table 1. The results of analysis of variance (ANOVA) for the significance of the linear, quadratic, and interaction terms of four independent parameters on the emulsifying activity were shown in Table 2.

**Table 2:** ANOVA for EAI response surface quadratic model.

Source	Sum of squares	DF	Mean square	F-value	*p*-value
Model	267.83	14	19.13	12.64	<0.0001*^1)^
X_1_	5.35	1	5.35	3.53	0.0812**
X_2_	3.38	1	3.38	2.23	0.1572**
X_3_	53.85	1	53.85	35.57	<0.0001*
X_4_	47.84	1	47.84	31.60	<0.0001*
X_1_ X_2_	0.076	1	0.076	0.050	0.8264**
X_1_ X_3_	9.52	1	9.52	6.29	0.0251*
X_1_ X_4_	2.18	1	2.18	1.44	0.2505**
X_2_ X_3_	2.92	1	2.92	1.93	0.1863**
X_2_ X_4_	0.000	1	0.000	0.000	1.000**
X_3_ X_4_	4.86	1	4.86	3.21	0.0947**
X_1_^2^	74.87	1	74.87	49.46	<0.0001*
X_2_^2^	82.09	1	82.09	54.23	<0.0001*
X_3_^2^	1.38	1	1.38	0.91	0.3560**
X_4_^2^	0.88	1	0.88	0.58	0.4580**
Residual	21.19	14	1.51		
Lack of fit	19.52	10	1.95	4.67	0.0753**
Pure error	1.67	4	0.42		
Cor total	289.02	28			

^1)^*significant.

**non-significant.

Analysis of variances indicates that the regression model for EAI is significant at *p* < 0.0001, while the lack of fit is not significant at *p* < 0.05. The coefficient of determination (R^2^) of 0.93 obtained for the model indicates a high degree of reliability for predictions of the EAI response. To investigate the effect of the hydrolysis temperature (X_1_), E/S ratio (X_2_), pH (X_3_), and hydrolysis time (X_4_) on the EAI response of wheat gluten hydrolysates, the following equation was used to quantify the response:

$$\text{Y}=55.56-0.67{\text{X}}_{1}+0.53{\text{X}}_{2}-2.12{\text{X}}_{3}+2.00{\text{X}}_{4}-0.14{\text{X}}_{1}{\text{X}}_{2}+1.54{\text{X}}_{1}{\text{X}}_{3}+0.74{\text{X}}_{1}{\text{X}}_{4}-0.86{\text{X}}_{2}{\text{X}}_{3}+0.00{\text{X}}_{2}{\text{X}}_{4}-1.10{\text{X}}_{3}{\text{X}}_{4}-3.40{\text{X}}_{1}{}^{2}-3.56{\text{X}}_{2}{}^{2}-0.46{\text{X}}_{3}{}^{2}-0.37{\text{X}}_{4}{}^{2}$$

Hydrolysis time and pH exhibited both linear effects on EAI, but no significant effect in the quadratic regions. Hydrolysis temperature and E/S ratios showed significant quadratic effects on EAI. In addition, hydrolysis temperature and pH had significant interactions. As shown in Table 2, pH was the most important variable affecting the EAI response.

**Fig 1:**
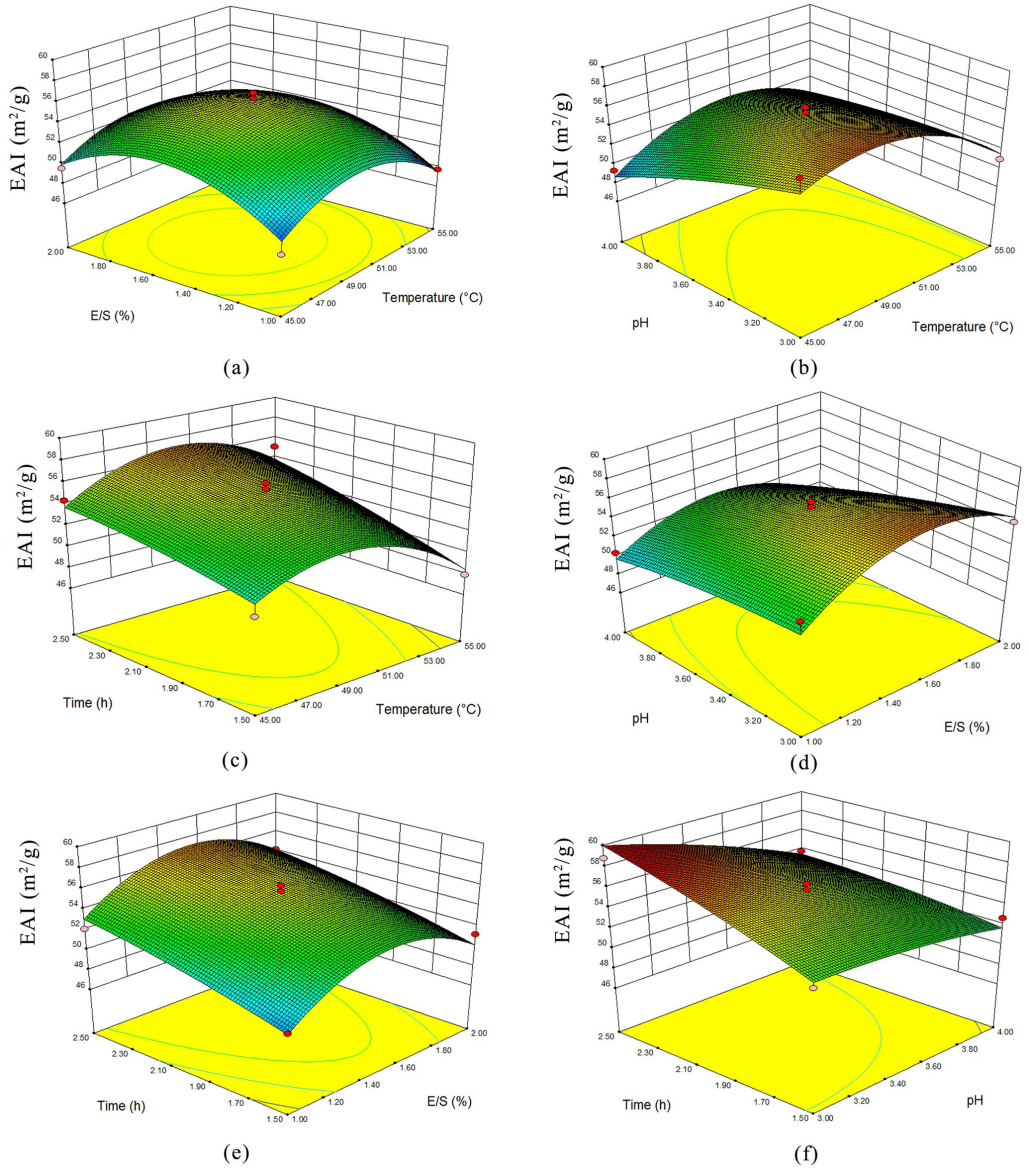
3D response surface plots of the interaction between factors. (a) effect of temperature and E/S ratio on the pH at 3.5 and time at 2.0 h; (b) effect of pH and temperature on the E/S at 1.5% and time at 2.0 h; (c) effect of time and temperature on the E/S at 1.5% and pH at 3.5; (d) effect of pH and E/S ratio on the time at 2.0 h and temperature at 50.0°C; (e) effect of time and E/S ratio on the pH at 3.5 and temperature at 50.0°C; (f) effect of time and pH on the E/S at 1.5% and temperature at 50.0°C.

The relationship between the parameters and the response was investigated using 3D response surface plots. From the interaction 3D surface between temperature and the other parameters (Fig 1a, 1b and 1c), it is clear that with the increase in temperature the EAI increased and then decreased after 50°C, which might be related to the inactivation of enzyme at high temperatures. E/S ratio showed the similar effect on EAI of hydrolysates (Fig 1a, 1d and 1e). The decrease of EAI at high E/S ratio could attribute to the saturation of the enzyme with the substrate. Fig 1b, 1d and 1e showed that EAI decreased with the increase of pH value. The effect of hydrolysis time suggested an opposite trend (Fig 1c, 1e and 1f), which may be related to higher DH.

According to the RSM analysis, the optimum reaction parameters were determined as follows: hydrolysis temperature, 48.9°C; E/S ratio, 1.60%; pH 3.0; hydrolysis time, 2.5 h. Thus, the EAI obtained at the optimum conditions was 60.25 m^2^/g, while the actual result was 58.65 m^2^/g as indicated by six verification experiments (results not shown). The differences between the actual and the estimated values were within 4%. The hydrolysate prepared in the optimized condition was used in the further analysis.

### Solubility

**Fig 2:**
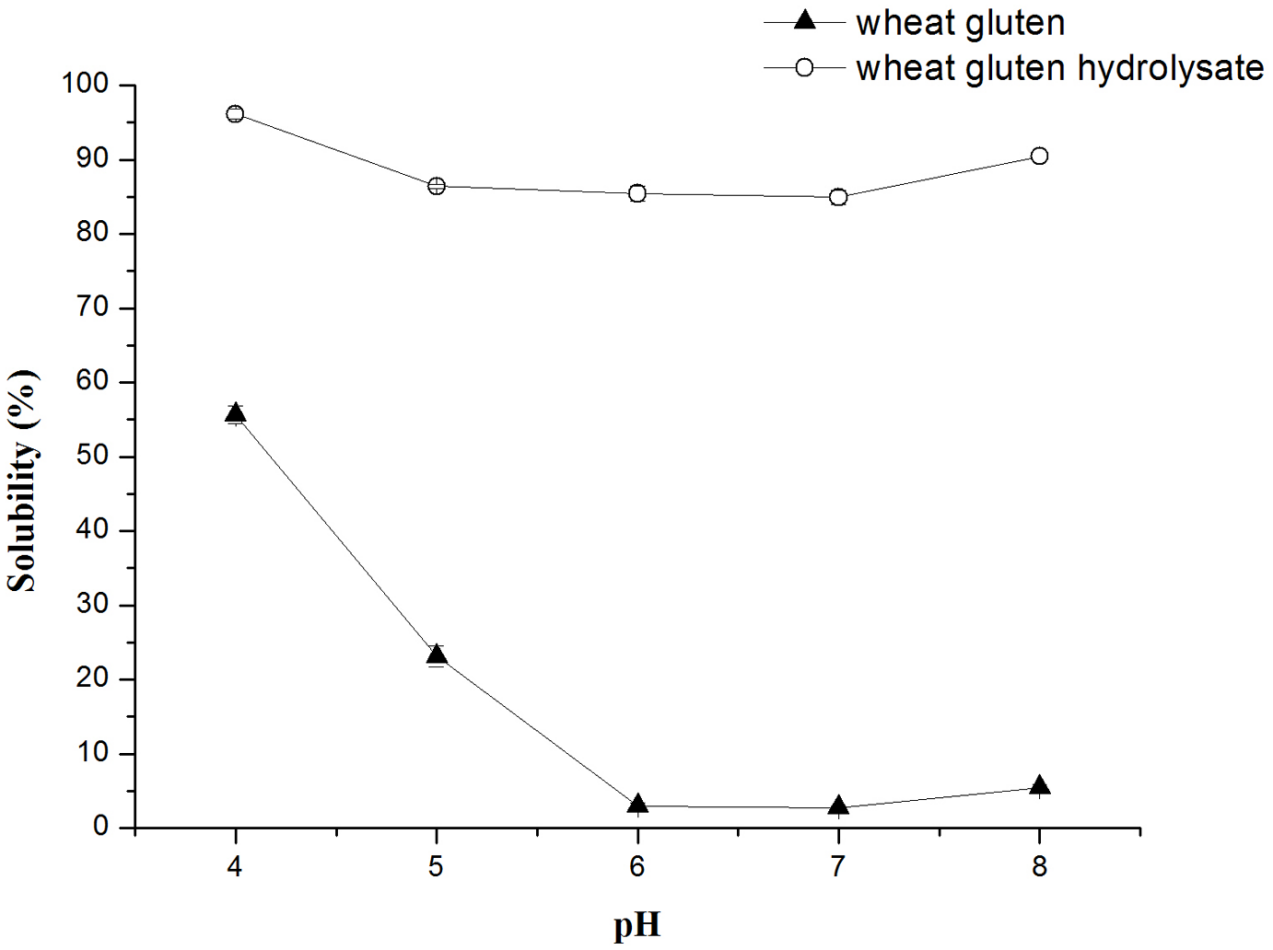
Solubility at different pH values. Solubility of the wheat gluten and its hydrolysate at different pH values.

As shown in Fig 2, the water solubility of wheat gluten was dramatically increased after hydrolysis. The pH-dependent solubility of the native wheat gluten showed a typical bell shape. However, the solubility of wheat gluten hydrolysate changed slightly with pH and no obvious isoelectric point could be observed. It was reported that the increased solubility of protein hydrolysates is due to the destruction of its secondary structure and the enzymatic release of smaller polypeptide units from the protein [19].

### Functional properties

It could be seen from Table 3 that the emulsifying activity was greatly improved from 18.05 to 58.65 m^2^/g after hydrolysis, but the emulsifying stability did not change much. Increasing the emulsifying activity without increasing the emulsifying stability shows the lack of relation between the two functional properties. The unfolding of wheat gluten’s globular structure and cleavage of peptides by enzyme could promote interaction between peptides and lipid, and help the anchoring of peptide molecules at the oil-water interface, leading to reduced interfacial tension and increased emulsifying activity [21]. However, the charged smaller peptides generated in enzymatic hydrolysis were unable to build thick films to stabilize the emulsion, as indicated by the low emulsifying stability. The contradictory results between emulsifying activity and stability was also reported by Mimouni *et al*. [6]. The emulsifying capacity and emulsifying stability of wheat gluten were increased by one fold after alcalase hydrolysis at DH of 5% by Kong *et al*. [8]. Wang *et al*. [9] found that the EAI of wheat gluten hydrolysate was improved by two folds compared to the original gluten, and the 50-K fraction of the hydrolysate showed a dramatical increase in EAI. However, the values for EAI and ESI so obtained apply only to the carefully defined system and may not be suitable to compare with other studies [12].

**Table 3:** Functional properties of wheat gluten and its hydrolysate.

Functional property	EAI (m^2^/g)	ESI (min)	Water holding capacity (g/g)	Oil holding capacity (g/g)
Wheat gluten	18.05 ± 3.03^a^	5.67 ± 0.13^a^	1.47 ± 0.06^a^	0.92 ± 0.05^a^
Wheat gluten hydrolysate	58.65 ± 0.33^b^	5.76 ± 0.10^a^	1.75 ± 0.03^b^	2.91 ± 0.22^b^

Results are presented as mean values ± standard deviation of 3 replicates. Different letters indicate significant difference (*p* < 0.05) between each parameter tested.

The water holding capacity of wheat gluten was increased slightly from 1.47 to 1.75 g/g, while the oil holding capacity increased significantly from 0.92 to 2.91 g/g after hydrolysis (Table 3). This might be related to enzymatic hydrolysis exposed more hydrophobic regions originally buried within the wheat gluten to the aqueous phase [22]. The values of oil holding capacity were higher than that of water holding capacity, indicating a higher level of hydrophobicity compared with hydrophilicity in the wheat gluten and its hydrolysate [23].

### Distribution of molecular weight

Molecular weight distributions of wheat gluten and its hydrolysate were shown in Table 4. The degree of hydrolysis of the wheat gluten hydrolysate was 4.94 ± 0.01%, in agreement with the results of Kong *et al*. [8]. A moderate degree of hydrolysis may contribute to significantly improvement of emulsifying activity. According to the molecular weight distribution, most peptides with high molecular weight (> 10 kDa) were enzymatically hydrolyzed into smaller peptides. The content of the fraction above 10 kDa decreased from 90% to 19% after hydrolysis, while that of small peptides below 2 kDa appeared, accounting for approximately 44% of the total hydrolysate.

**Table 4:** Molecular weight distribution profile of wheat gluten compared to its hydrolysate.

Molecular weight (kDa)	> 10	10 ~ 5	5 ~ 2	2 ~ 1	1 ~ 0.2	< 0.2	DH (%)
Wheat gluten	90.53 ± 0.20	8.19 ± 0.17	1.28 ± 0.03	0	0	0	0
Wheat gluten hydrolysate	19.49 ± 0.58	16.86 ± 0.81	19.58 ± 0.08	12.65 ± 0.42	28.33 ± 0.51	3.09 ± 0.23	4.94 ± 0.01

### Secondary structure change

**Fig 3:**
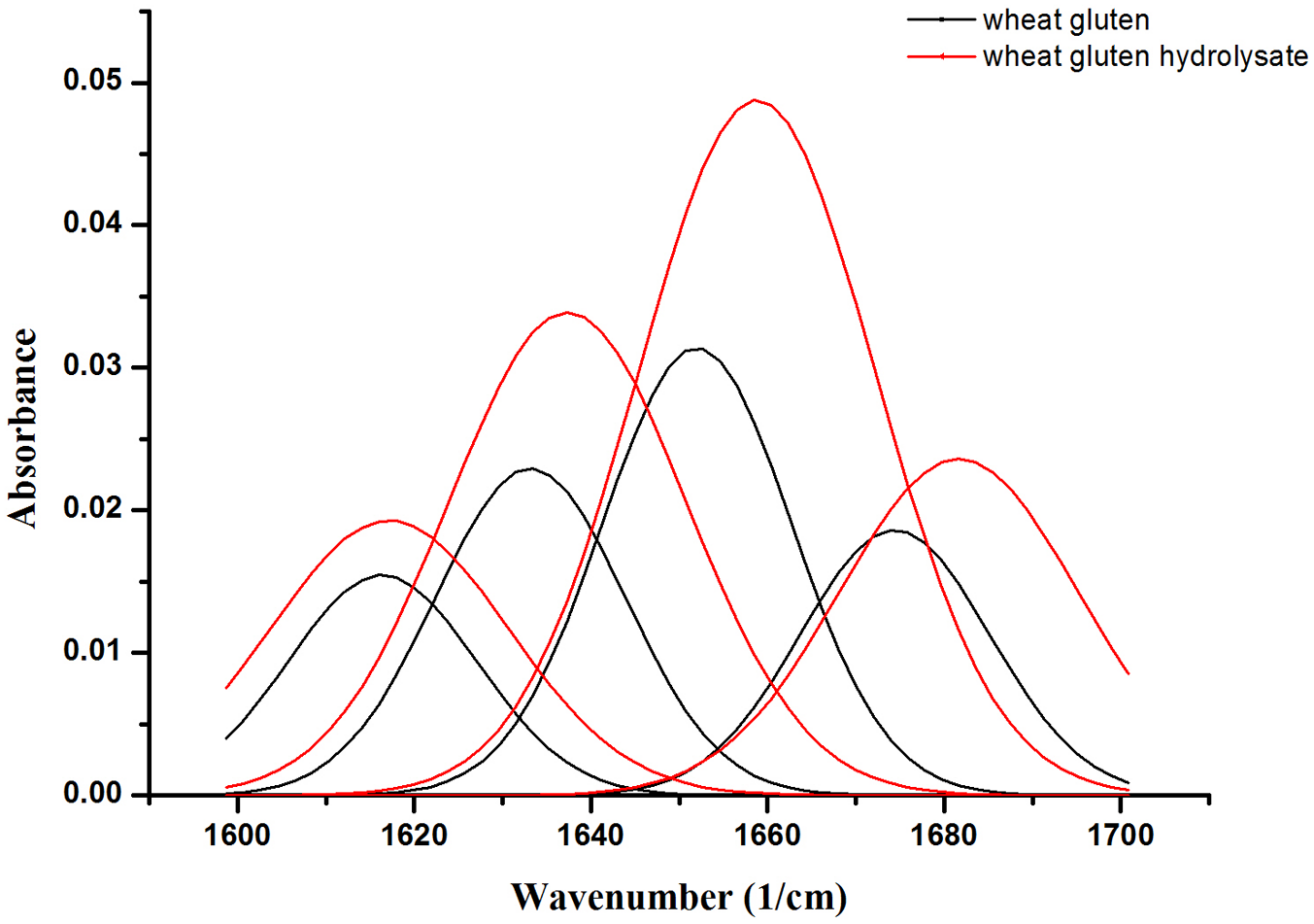
Curve fitting of deconvolved amide I bands. Curve fitting of deconvolved amide I bands of wheat gluten and its hydrolysate assuming Gaussian band shapes for deconvolved components.

The conformational change of wheat gluten was studied by FTIR spectra. The amide I band of polypeptides has long been known to be sensitive to secondary structure and has caused considerable interest in the understanding of the structure-spectrum relationship [14]. By analyzing the amide I region (1600–1700 cm^-1^) of FTIR spectra, the curve fittings of amide I bands of wheat gluten and its hydrolysate were shown in Fig 3, and the contents of secondary structure components were calculated (Table 5). According to the previous reports [14, 24], secondary structures were assigned as follows: α-helix (1648–1667 cm^-1^), β-sheet (1623–1641 cm^-1^), and turns (1670–1687 cm^-1^). The bands at 1617 cm^-1^ could be a combination of intermolecular β-sheet structure and the amino acid side chain residue vibration [16]. A common problem of the assignment occurred in studies is the overlap of α-helix and random structure, which could explain the high ratio of α-helix [14].

**Table 5:** Assignment of secondary structure and the content of corresponding structure of wheat gluten and its hydrolysate.

	Wavenumber (cm^-1^)	Structure	Secondary structure content (%)
Wheat gluten	1619	β-sheet	40.23 ± 0.34^a^
	1633	β-sheet	
	1651	α-helix	38.42 ± 0.30^a^
	1675	Turns	21.35 ± 0.44^a^
Wheat gluten hydrolysate	1617	β-sheet	42.03 ± 0.10^b^
	1637	β-sheet	
	1658	α-helix	39.81 ± 0.88^a^
	1679	Turns	18.16 ± 0.23^b^

Results are presented as mean values ± standard deviation of 3 replicates. Different letters indicate significant difference (*p* < 0.05) between each parameter tested.

As shown in Fig 3, a more pronounced shoulder around 1618 cm^-1^ was observed in wheat gluten hydrolysate, indicating an increase in intermolecular β-sheet structures. The absorption corresponding to intramolecular antiparallel β-sheet (1633 cm^-1^) of the native gluten shifted to 1637 cm^-1^ after hydrolysis. A correlation has been proposed that the chain length of β-sheets and the number of chains have effect on the wavenumber of the bands [25]. The band assigned as α-helix shifted from 1651 cm^-1^ to 1658 cm^-1^ after hydrolysis. Only the longer helix gives a calculated spectrum with the ‘typical’ main band near 1650 cm^-1^, which suggests that α-helix has been cleaved into shorter helix during hydrolysis [26]. The dependence of the band position on the helix length seemed to be correctly predicted by theory since myoglobin absorbs near 1655 cm^-1^ and tropomyosin with longer helix near 1646 cm^-1^ [27]. The turns structure was expected to absorb between 1700 cm^-1^ to 1630 cm^-1^, depending on the type of turn and on the dihedral angles. After enzymatic hydrolysis, the band assigned to turns shifted 4 cm^-1^.

The total β-sheet content of wheat gluten was increased from 40.23 ± 0.34% to 42.03 ± 0.10% after hydrolysis, while the turns content was decreased from 21.35 ± 0.44% to 18.16± 0.23%. In globular proteins the β-sheet are usually quite short, and the sheets often involve only a limited number of chains, sometimes as few as two or three [15]. Due to enzymatic cleavage of peptide chains, the globular structure of gluten has been unfolded and the ratio of turns decreased, hence the ratio of β-sheet increased and longer β-sheet chains generated [14].

### Tertiary structure change

The SH content was decreased from 8.88 to 6.17 μmol/g after enzymatic hydrolysis, while the SS content was increased from 10.67 to 15.71 μmol/g (Table 6). The results indicated that the exposed SH groups were also reactive to be oxidized to SS bonds at acidic pH on heating, resulting in an increase of SS bonds in hydrolyzed gluten [16, 28]. It has been suggested that alcalase and esperase hydrolysis of soy protein led to a decrease in the amounts of SH and increase in the amounts of SS [29]. And Zhao *et al*. [16] found that the SS content of wheat gluten increased as deamidation levels increased.

**Table 6:** Content of disulphide bonds (SS) and sulfhydryl groups (SH) in wheat gluten and its hydrolysate.

	SH content (μmol/g)	SS content (μmol/g)
Wheat gluten	8.88 ± 0.09^a^	10.67 ± 0.61^a^
Wheat gluten hydrolysate	6.17 ± 0.14^b^	15.71 ± 0.77^b^

Results are presented as mean values ± standard deviation of 3 replicates. Different letters indicate significant difference (*p* < 0.05) between each parameter tested.

Gluten comprises monomeric gladins and polymeric glutenins consisting of high and low molecular weight subunits (HMW and LMW). It is thought that the glutenin polymers are stabilized by interchain disulfide bonds formed between cysteine residues located in the LMW and HMW subunits and that the individual polymers interact with other glutenin polymers and gliadin proteins by non-covalent interactions [30]. Thus, it can be inferred that in the enzymatic hydrolysis some SS bonds were broken to unfold the globular structure of gluten, and new SS bonds were generated between both newly generated SH and original unpaired SH to stabilize the structure of smaller peptides.

## Conclusions

A temperature of 48.9°C, E/S ratio of 1.60%, pH 3.0, hydrolysis time of 2.5 h were found to be the optimum condition to obtain the acid protease (from *Aspergillus usamii*) hydrolyzed wheat gluten with improved functional properties. The water solubility of wheat gluten was greatly improved, and became independent of pH value. The water and oil holding capacity were improved by hydrolysis as well. Enzymatic hydrolysis degraded most of the peptides above 10 kDa into much smaller peptides. With the secondary and tertiary structure change during the enzymatic hydrolysis, a more extensional structure was formed, leading to the increase in functional properties.

## Supporting Information

S1 Table Factors for the response surface Box-Behnken design. Temperature, E/S ratio, pH, and hydrolysis time at three levels for the response surface Box-Behnken design.
(DOCX)
