# Supplementary material for: Improvement of Functional Properties of Wheat Gluten Using Acid Protease from Aspergillus usamii
Source: PLoS One. 2016 Jul 28;11(7):e0160101. doi: 10.1371/journal.pone.0160101 (PMC4965038; doi:10.1371/journal.pone.0160101)
Supplement: S1 Table — Temperature, E/S ratio, pH, and hydrolysis time at three levels for the response surface Box-Behnken design. (DOCX) [file pone.0160101.s001.docx]

S1 Table. Temperature, E/S ratio, pH, and hydrolysis time at three levels for the response surface Box-Behnken design.

| Factor | Coded symbol | Levels | | |
| --- | --- | --- | --- | --- |
|  |  | -1 | 0 | 1 |
| Temperature (°C) | X_1_ | 45 | 50 | 55 |
| E/S ratio (%) | X_2_ | 1.0 | 1.5 | 2.0 |
| pH | X_3_ | 3.0 | 3.5 | 4.0 |
| Hydrolysis time (h) | X_4_ | 1.5 | 2.0 | 2.5 |
